# Genomic Resources for Water Yam (*Dioscorea alata* L.): Analyses of EST-Sequences, *De Novo* Sequencing and GBS Libraries

**DOI:** 10.1371/journal.pone.0134031

**Published:** 2015-07-29

**Authors:** Christopher A. Saski, Ranjana Bhattacharjee, Brian E. Scheffler, Robert Asiedu

**Affiliations:** 1 Institute for Translational Genomics, Genomics and Computational Biology Laboratory, Clemson University, Clemson, SC, United States of America; 2 Bioscience Center, International Institute of Tropical Agriculture, Ibadan, PMB 5320, Nigeria; 3 Genomics and Bioinformatics Research Unit, USDA-ARS, Stoneville, MS, United States of America; National Institute of Plant Genome Research, INDIA

## Abstract

The reducing cost and rapid progress in next-generation sequencing techniques coupled with high performance computational approaches have resulted in large-scale discovery of advanced genomic resources in several model and non-model plant species. Yam (*Dioscorea* spp.) is a major food and cash crop in many countries but research efforts have been limited to understand the genetics and generate genomic information for the crop. The availability of a large number of genomic resources including genome-wide molecular markers will accelerate the breeding efforts and application of genomic selection in yams. In the present study, several methods including expressed sequence tags (EST)-sequencing, *de novo* sequencing, and genotyping-by-sequencing (GBS) profiles on two yam (*Dioscorea alata* L.) genotypes (TDa 95/00328 and TDa 95-310) was performed to generate genomic resources for use in its improvement programs. This includes a comprehensive set of EST-SSRs, genomic SSRs, whole genome SNPs, and reduced representation SNPs. A total of 1,152 EST-SSRs were developed from >40,000 EST-sequences generated from the two genotypes. A set of 388 EST-SSRs were validated as polymorphic showing a polymorphism rate of 34% when tested on two diverse parents targeted for anthracnose disease. In addition, approximately 40X *de novo* whole genome sequence coverage was generated for each of the two genotypes, and a total of 18,584 and 15,952 genomic SSRs were identified for TDa 95/00328 and TDa 95-310, respectively. A custom made pipeline resulted in the selection of 573 genomic SSRs common across the two genotypes, of which only eight failed, 478 being polymorphic and 62 monomorphic indicating a polymorphic rate of 83.5%. Additionally, 288,505 high quality SNPs were also identified between these two genotypes. Genotyping by sequencing reads on these two genotypes also revealed 36,790 overlapping SNP positions that are distributed throughout the genome. Our efforts in using different approaches in generating genomic resources provides a non-biased glimpse into the publicly available EST-sequences, yam genome, and GBS profiles with affirmation that the genomic complexity can be methodically unraveled and constitute a critical foundation for future studies in linkage mapping, germplasm analysis, and predictive breeding.

## Introduction

Yam (*Dioscorea* spp.) a multi-species, polyploid and vegetatively propagated crop, is an economically important staple food crop in West and Central Africa, the Pacific and Caribbean Islands, and parts of some Asian and Latin American countries. With the exponential global population growth and the consequent rise in the demand for food, efforts to improve yam breeding are urgent. There has been rapid development of DNA informed breeding techniques, e.g. marker-assisted breeding, and ‘genomic selection’, through which model agricultural crops such as maize, rice, wheat, and soybean have seen significant genetic gains. The ability to associate genotype with phenotype in a population-specific context is also providing a translational interface between basic research scientists and plant breeders. The advancement of yam breeding programs to contemporary levels for designing new genotypes with resistance or tolerance to biotic and abiotic stresses has been significantly constrained because of the lack of systematic knowledge and understanding of the genetics and genomics of the crop. There are limited genomic or genetic resources, and no mapped genetic markers that can be directly applied to breeding for any species of yam.

In an earlier effort led by Mignouna et al [1], a genetic linkage map for tetraploid white yam (*Dioscorea rotundata* Poir.) and water yam (*D*. *alata* L.) were constructed based on 341 and 469 co-dominantly scored amplified fragment length polymorphism (AFLP) markers, respectively using intraspecific F1 segregating populations. These QTL mapping efforts revealed one AFLP marker E-14/M52-307 located on linkage group 2 that was associated with anthracnose resistance, explaining 10% of the total phenotypic variance in *D*. *alata*. Similarly, Petro et al [2] developed an intraspecific genetic linkage map of water yam (*D*. *alata* L.) using 523 AFLP markers and a total of nine QTL (s) were identified for anthracnose disease. Prospects for detecting additional QTLs and applying marker-assisted selection in *D*. *alata* breeding appears promising, however this is subject to the development of additional molecular markers in the crop for better and wider genome coverage.

The availability of a large number of molecular markers is a prerequisite for identifying informative ones for genetic mapping or associating them to traits of interest, thus advancing breeding strategies like marker assisted selection or genomic selection [2, 3]. Over the years, 25 polymorphic genomic SSR loci from *D*. *alata*, *D*. *abyssinica*, *D*. *praehensilis* [4] and 8 genomic SSRs from *D*. *trifida* [5] were identified. Several of these markers are not transferable to species of other *Dioscorea* sections. The availability of new generation markers, such as genomic SSRs, EST-SSRs and SNPs, will provide tools for wider compatibility across different species, genetic analysis of traits of economic importance, and for marker-assisted selection in yam breeding programs.

Efforts reported here utilized (ESTs generated from two *D*. *alata* genotypes (TDa 95/00328 and TDa 95–310) in a previous study [6] to identify EST-SSRs. Thousands (≥ 40,000) of expressed sequence tags (ESTs) were developed from these two genotypes, used as parents by Mignouna et al [7] for anthracnose disease. We also used next-generation sequencing technologies, such as *de novo* sequencing and GBS of the same genotypes to generate genomic SSRs and SNPs. This study aims to generate additional genomic and molecular resources in *D*. *alata* from different ongoing initiatives.

## Materials and Methods

### Plant DNA Source and Isolation

Two diploid *D*. *alata* genotypes showing differential reaction to anthracnose disease [8], were chosen as parents for crossing and development of a mapping population. The female parent (TDa 95/00328) (P1) is resistant to anthracnose disease while the male parent (TDa 95–310) (P2) is the susceptible genotype. About 1 g fully expanded young leaf samples from the parents were harvested at IITA-Ibadan, Nigeria. Leaf samples were freeze-dried and sent to USDA-ARS Stoneville and Clemson University for DNA extraction and genomic analysis. A modified CTAB extraction method was used for DNA extraction. DNA samples were run on 1% w/v agarose gel along with size standards to assess the quality and quantity. For GBS quality DNA, the methods of Brunner et al., [9] were modified where a total of 30mg of wet tissue was ground in LN2, 5% PVP (MW 340,000) was supplemented in each extraction tube, and incubated at 65°C for 30 minutes, mixing gently every 10 minutes. The extraction solution was then centrifuged, extracted two times with chloroform, and cleaned by isoproponal precipitation.

### Identification of EST-SSRs from available EST-sequences

A total of 40,000 EST-sequences from three cDNA libraries viz., 87-01091-33, A328-33, and B310-33 were assembled using the CAP3 program, on default settings, to reduce redundancy between libraries. The resulting contigs and singletons were screened for SSRs using SSRFinder (http://www.maizemap.org/bioinformatics/SSRFINDER/README.ssrfinder), which locates SSRs, designs primer pairs to amplify the regions containing the SSRs, and removes primer pairs that are not unique. A BLASTX was performed against the GeneBank non-redundant database version 181, with the parameter set to only return results where the maximum expected value was less than or equal to 0.001. Using Excel “lookup” function, the position of each sequence’s repeat was lined up with the BLASTX query begin/end positions. Then, using Excel “if” function, each SSR was labeled as being either “before,” “after,” or “within” the region of homology. To further improve the probability of selecting quality SSRs, the BLASTX results were screened for terms like ribosomal, retro-element, GAG protein, chloroplast, and mitochondria which were subsequently eliminated from further analysis. Based on the above criteria 1152 SSRs and associated primers were selected and categorized as follows:

1. BLASTX homology outside of the region containing an SSR (*746 primers*)
2. No BLAST homology found in entire sequence (*203 primers*)
3. BLAST homology to a region containing an SSR (*203 primers*)

### DNA Sequence Generation

A total of four sequencing libraries, two libraries from genomic DNA of each genotype, were prepared as described in the Epicentre Biotechnologies (Madison, WI) original fragmentation protocol with HMW Buffer (Nextera DNA Sample Prep Kit; Illumina-compatible; Cat. Nos. GA091120 and GABC0950). For sequencing library size selection to remove fragments below 300 bp, Agencourt AMPure XP beads (Product number A63880, Beckman Coulter, Inc, Danvers, MA) were used at a ratio of 0.7X volume/volume of library preparation. Completed libraries were assayed for size distribution on an Agilent 2100 Bioanalyzer with a High Sensitivity DNA LabChip (Product number 5067–4626, Agilent Technologies, Santa Clara, CA) and for concentration with an Illumina library quantification kit (Product number KK4854, Kapa Biosystems, Inc, Woburn, MA) on a qPCR instrument (LightCycler 480, Roche Applied Science, Indianapolis, IN). The libraries were clustered via cBot (Illumina, Inc, San Diego, CA), with each library on a single lane of a TruSeq PE Cluster Kit v3 paired-end flowcell (Product number PE-401-3001, Illumina, Inc, San Diego, CA). An Illumina PhiX v3 control library (Illumina Product number FC-110-3001) spike-in of <1% (v/v) was included in each lane to allow sequencing error rate calculation. Paired-end 2 x100 bp sequencing was carried out on an Illumina HiSeq 2000 with a total of 209 cycles of TruSeq SBS Kit v3 (Product number FC-401-3001, Illumina, Inc, San Diego, CA) chemistry, with the sequencing primer set included in the original Epicentre Biotechnologies Nextera DNA Sample Prep Kit.

### Genome Assembly

For assembly, the commercial product Genomic Workbench from CLCbio (Cambridge, MA), using default parameters for trimming and assembly, was employed. The HiSeq 2000 sequences for each parental genotype were submitted independently to CLC Genomic Workbench operating on a high memory node with 48 core processors and 512G RAM dedicated to each assembly.

### SSR Selection Pipeline from Genomic DNA Sequences

**Fig 1:**
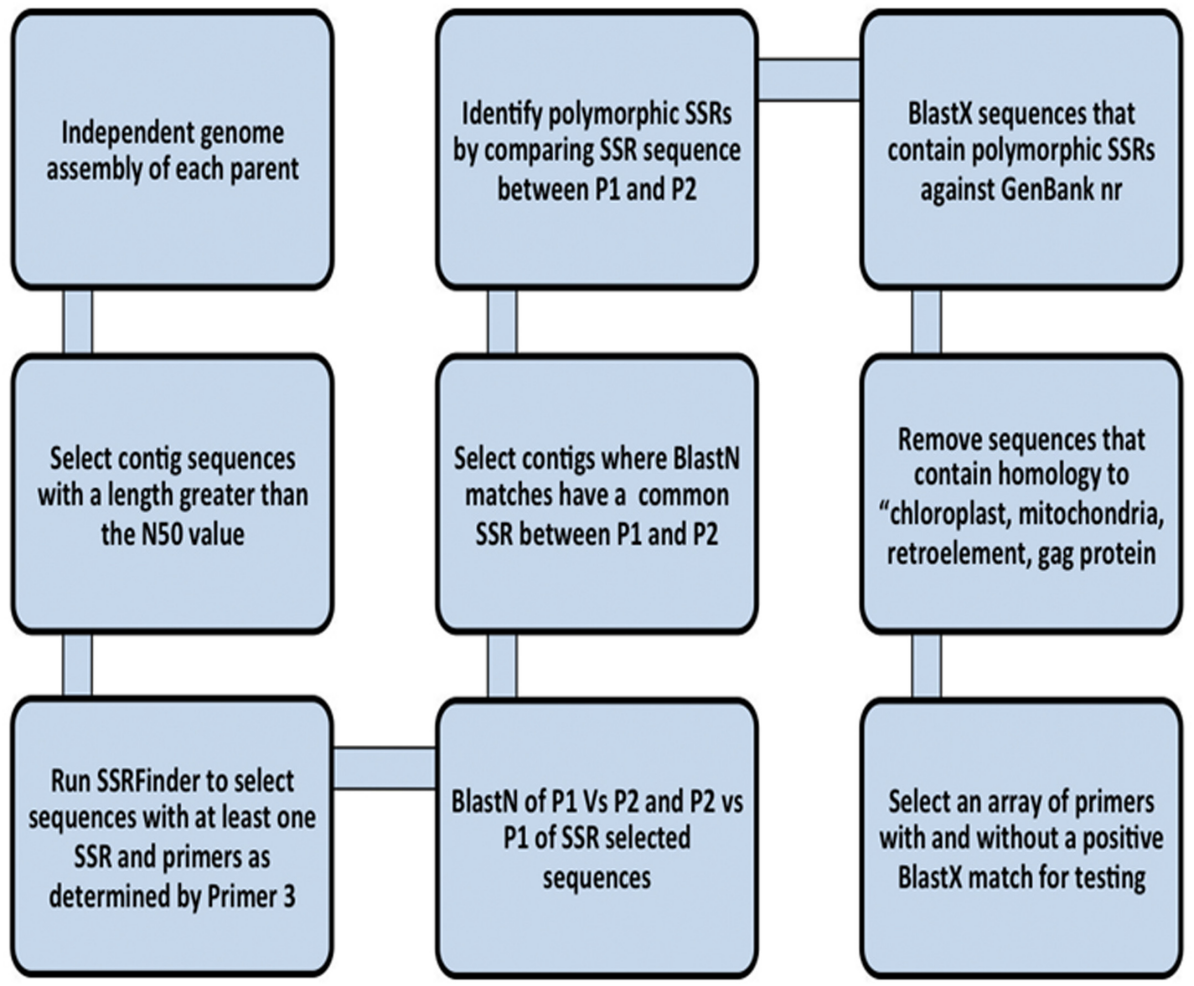
Genomic SSR discovery pipeline and primer design. The workflow starts with an independent assembly of each genotype and enrichment for sequence contigs with a length greater than the N50 value. Next, SSRFinder is used to find SSRs and design primers. Possible polymorphisms are detected and screened against the nr database (GenBank) and a priority given to non-repetitive or organellar sequences.

The overall pipeline has multiple steps which are outlined in Fig 1. The first step was an independent *de novo* assembly of genomic sequences from each parental genotype. As this resulted in a large number of contigs, a data reduction strategy was used where only contigs that were greater in size than the N50 value were selected (S1 Table). The selected sequences were processed through SSRFinder (http://www.maizemap.org/bioinformatics/SSRFINDER/README.ssrfinder) using default parameters and a minimum repeat length of 20 bp. Sequences that had at least one SSR and a suitable pair of primers for amplification were selected for a bi-directional BlastN (maximum E-value of e-9) [10, 11] search where the selected sequences of P1 were used as the query sequences against a database composed only of P2 sequences and the reverse was also processed. The BlastN search only identified those sequences that were shared between the two genotypes, it was then necessary to use a combination of Excel and PERL scripts that identified BlastN hits that shared a common SSR between P1 and P2 and those that were predicted to be polymorphic based on the length of the SSR in each of the genotypes. Those DNA sequences that were polymorphic were used in a BlastX (maximum E-value of e-9) [10, 11] search against GenBank nr protein database. Sequences with the descriptor having the following terms were eliminated for further processing: chloroplast, mitochondria, retroelement, or gag protein. This step was done to limit the number of SSRs located in repetitive or organelle DNA sequences. For validation testing, primer pairs were selected only from di- and tri-nucleotide repeat SSRs.

### SSR Genotyping

Primers for both genomic and EST-SSRs were ordered from the firm Integrated DNA Technologies (Coralville, IA), normalized at 6 nmols in a 96 well plate. For genotyping, a tailing method was used to add a florescently labeled tag to the final PCR product as a third primer homologous to the tail. Forward primers were 5′ tailed with the sequence 5′-CAGTTTTCCCAGTCACGAC-3′ and the third tailed primer was Primer 5′-CAGTTTTCCCAGTCACG AC-3′ labeled with 6-carboxy-fluorescein. The reverse primers were tailed at the 5′ end with the sequence 5′-GTTT-3′ to promote non-template adenylation [12]. The 5 μl PCR reaction contained 10 ng genomic DNA, 0.4 μl Titanium Taq DNA Polymerase (Clontech, Mountain View, CA), 0.5 μl Titanium buffer, 0.04 μl dNTP (25mM), 0.8 μl FAM labeled primer (05 pmol/μl), 0.4 pmol of forward primer and 1.2 pmol reverse primer). The reaction was run on a BioRad thermocycler (Hercules, CA) at 95°C for one min, 60°C for one min (two cycles), 95°C for 30 s, 60°C for 30 s, 68°C for 30 s (27 cycles), and a final extension at 68°C for four min. Fluorescently labeled PCR fragments were analyzed on an ABI 3730XL DNA Analyzer with ROX 500 size standard and data-processed using GeneMapper Version 5.0 (all three from Applied Biosystems, Foster City, CA). Each primer pair was tested on P1 and P2 and the data compared to determine the number of primer pairs that amplified, the allele number and size, and their polymorphic rate.

### SNP Determination between P1 and P2

Genome-wide SNPs were determined by first aligning the sequence reads from P1 to the P2 reference assembly with the Bowtie2 short read mapper [13], with the very-sensitive-local flag, to produce a SAM file. The SAM file was converted to BAM, sorted, and indexed, with the samtools software suite (http://samtools.sourceforge.net). An mpileup file was created and converted to a.bcf file with the samtools software suite (http://samtools.sourceforge.net). SNPs were called with the bcftools software tools (http://samtools.sourceforge.net) with the—bvcg parameters and piped into the vcfutils.pl script (http://samtools.sourceforge.net) to produce a final variant call file (.vcf). The vcf file was filtered for sites that contain at least 10 sequence reads and a mapping quality of 30 with the vcftools software suite [14, 15].

### GBS-SNPs between P1 and P2

Genotying by sequencing libraries were prepared from 200ng of purified gDNA with the *Pst*1 enzyme for genomic selection closely following the methods of Elshire et al [16]. Sequence data was collected on a HiSeq2500 for each GBS parent. Raw sequence reads were demultiplexed and collapsed into unique tags with the Tassel 4.0 Standalone software package [17, 18]. Unique tags for each parent were aligned to the P2 reference genome assembly with the bowtie2 [13] software using the—very-sensitive-local flag. The SAM file was converted to BAM, sorted, and indexed, with the samtools software suite (http://samtools.sourceforge.net). Overlap between the bam files was calculated by extracting the coverage from each bamfile into a bedgraph format with the bedtools software [19, 20] and the genomeCoverage command. A union bedgraph was created that contains the overlap information by using the unionBedGraphs command and the bedtools software [19, 20]. Quality statistics were determined for each GBS BAM file with the qualimap software [21].

## Results

### EST-SSRs from EST-sequences generated from *D*. *alata* genotypes

A total of 1,152 EST-SSRs were developed from 40,000 ESTs generated from two *D*. *alata* genotypes used as parents to generate a mapping population for anthracnose disease (resistant parent, TDa 95/00328 and susceptible parent, TDa 95–310) [8]. The rate of polymorphism detected with EST-SSRs was relatively low. Out of 1,152 EST-SSRs, hundred and two failed to produce a PCR product and 466 were monomorphic (Table 1). A total of 584 EST-SSRs were found to be polymorphic (Table 1) with 445 SSRs produced a single product (one locus), 70 with two products (possibly an indication of duplicated genes) and sixty-nine multi-allelic indicating the polyploid nature of the crop. Based on quality of PCR products and allelic patterns, 388 SSRs out of 445 were identified as good quality polymorphic SSRs, of which 152 were considered high quality, 210 as possible to use and 26 as undetermined quality for mapping purposes. Repeat motifs of 1152 EST-SSRs ranged from di- to hepta-nucleotides, with a dominant representation (65.8%) of tri-nucleotide repeats (Table 1). A complete summary of the EST-SSR data, including SSR-sequence, repeat motifs, product range, and primer sequences is provided in S1 Table.

**Table 1:** Repeat motifs of EST-SSRs and their numbers.

Markers	Total	Di's	Tri's	Tetra’s	Penta’s	Hexa’s	Hepta’s
**Polymorphic**	584	176	327	42	11	25	3
**Monomorphic**	466	56	357	33	8	12	0
**No amplification**	102	23	74	2	0	3	0
**Total**	1152	255	758	77	19	40	3
**Percent**		22.14%	65.80%	6.68%	1.65%	3.47%	0.26%

### Genomic SSRs from *de novo* sequencing of the same genotypes

Whole genome shotgun sequences were generated on a HiSeq 2000 for TDa 95/00328 and TDa 95–310 using two lanes of 2X100bp paired end reads for each genotype. Depending on the real genome size, quality of the DNA, etc., an estimated 40X genome coverage was achieved. The number of reads for the susceptible genotype (P2) (TDa 95–310) were 849,524,270 and that of the resistant genotype (P1) (TDa 95/00328) were 530,269,368. The results from CLC Bio program showed limited scaffolding and this was expected due to the use of paired-end sequencing of the genotypes. It is noteworthy that N25-75 results were slightly skewed, but considering that leaf tissues were used as DNA source, large chloroplast or mitochondrial contigs were also expected to be present in the assembly. Nonetheless, the assembled data is of sufficient quality to be used to gain insight into gene spacing, and can be used as a sequence database for BLAST searches to find gene (s) of interest. The resulting assembly covered a total of 428,947,971 bps (excluding scaffolded regions) with N50 of 858 for P1 and 374,652,491 bps (excluding scaffolded regions) with N50 of 1,171 for P2 (S2 Table), which is almost similar to that estimated for *D*. *rotundata*, about 540 Mb (unpublished). Contig sizes ranged from 100bp to 95kb with an average of ~450bp between both genotypes (S2 Table). The nucleotide content values for each genotype are almost identical with a A+T to G+C ratio of 64% to 36% (S3 Table). These G+C values suggest that a high quality yam genome assembly based on traditional whole genome shotgun approaches predominantly composed of Illumina generated data should not be influenced by G+C coverage bias [8, 22].

Additionally, polymorphic genomic SSRs were identified using a customized design (Fig 1). For P1, a total of 18,584 SSRs were identified, and the program failed to design primers for 4,801 SSRs. Similarly, a total of 15,952 SSRs, of which 3,866 were not suitable for primer design, were idenfied in P2. For P1, 13,783 primed SSRs from 11,871 sequences while 12,086 primed SSRs resulted from 9,903 sequences in P2, indicating that one, or more than one, SSRs are primed on the same sequence. Next, two BlastN searches between the two genotypes were performed where one search used P1 to query the P2 database, and vice versa. The matching of P1 with P2 resulted in 2,041 SSRs that matched but were monomorphic, 572 matched and were polymorphic, and 503 of P1 did not match with those of P2 (Table 2). A reciprocal comparison showed that 2,027 SSRs matched but were monomorphic, 573 matched and were polymorphic, and 515 of P2 did not match with those of P1 (Table 2). The resulting 573 SSRs were tested for polymorphism across both parents, and a set of 384 putative polymorphic genomic SSRs from the two *D*. *alata* genotypes were identified. The repeat motifs of 384 identified SSRs ranged from di- to hepta-nucleotide repeats with the dominant representation (36.98%) of trinucleotide repeats as observed in EST-SSRs (Table 3). A comparison of the predictive polymorphic rate among EST- and genomic SSRs showed a higher polymorphism to an extent of 83.51% among genomic SSRs with the failure of only 8 SSRs (Table 4). The details of SSR marker sequences, repeat motifs, and allelic range is provided in S4 Table.

**Table 2:** Genomic SSR discovery in two parental lines.

SSR Pattern Type	P1 vs. P2	P2 vs P1
*Monomorphic*	2,041	2,027
*Polymorphic*	572	573
*No Match*	503	503

**Table 3:** Repeat motifs of genomic SSRs and their numbers.

Markers	Total	Di's	Tri's	Tetra’s	Penta’s	Hexa’s	Hepta’s
**Polymorphic**	339	117	134	64	17	6	1
**Monomorphic**	37	10	7	8	11	1	
**No amplification**	8	5	1	2			
**Total**	384	132	142	74	28	7	1
**Percent**		34.38%	36.98%	19.27%	7.29%	1.82%	0.26%

**Table 4:** Comparison between EST- and genomic derived SSRs.

SSR Pattern Type	EST-SSR	Genomic SSR
*Monomorphic*	530	62
*Polymorphic*	388	314
*Failed*	103	8
*% polymorphic*	42	84
*% failure*	10	2

### Genomic SNPs and GBS profiles of the same genotypes

**Fig 2:**
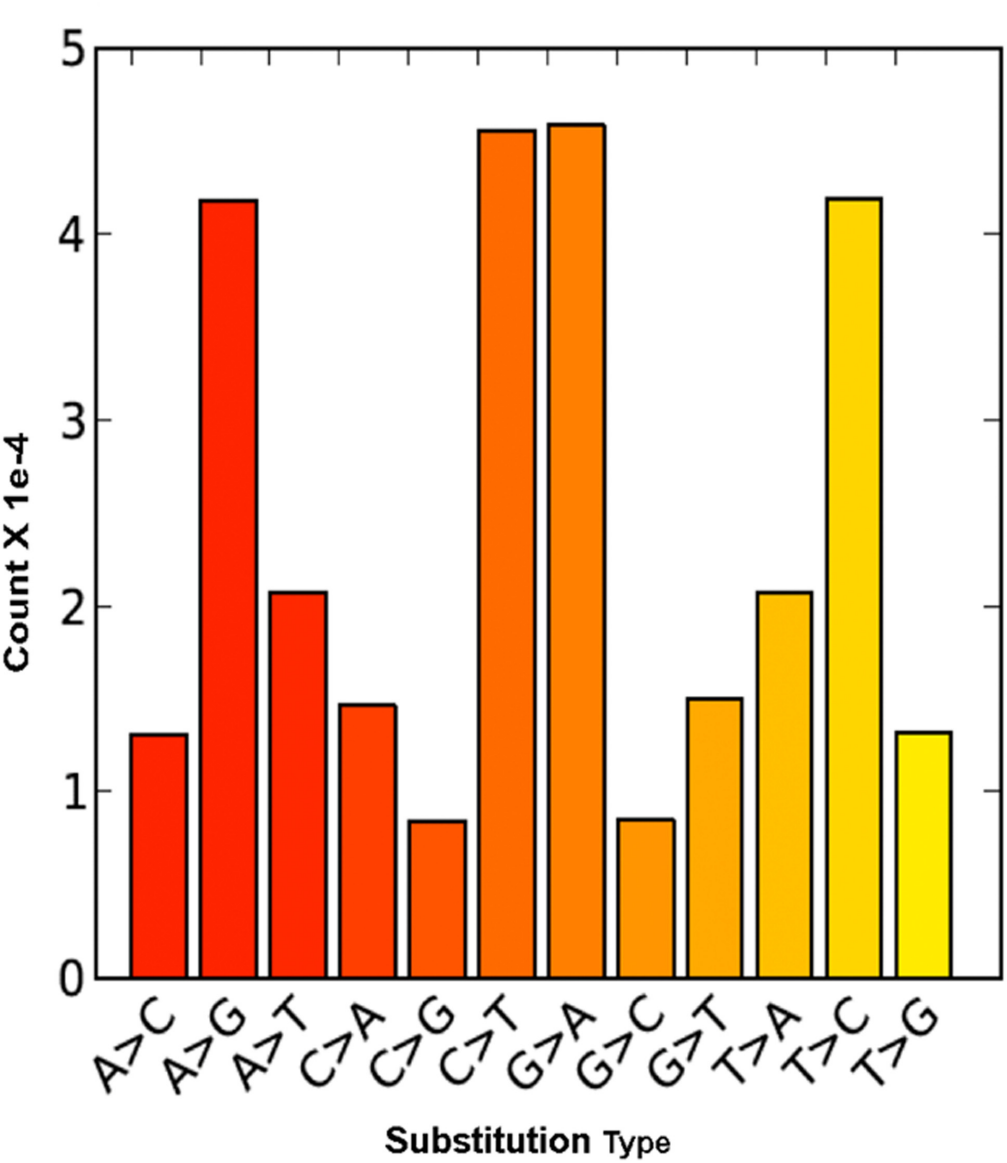
Nucleotide substitute distribution type and counts. The SNP nucleotide substitution type and count as determined between both genotypes.

Genomic SNPs were determined by first aligning the 48 million reads of P1 to the reference assembly of P2 (765,175 sequences); which resulted in an overall alignment rate of 76.51%, of which, 46.06% are unique read alignments (S5 Table). Using several Bioinformatic tools, a total of ~1.8 M SNPs, 155k InDels, and ~10k multiallelic SNP sites were identified. After filtering for sites that have at least 10 sequences and a mapping quality score of 30, the final variant set consisted of 288,505 SNPs, 30,253 InDels, and only 693 multiallelic positions. The most dominant SNP conversions are G→A and C→T, comprising 30% of the variant set. Next, A→T and T→A are close to 15% in abundance, while C→G conversions were the least represented, ~3% (Fig 2).

**Fig 3:**
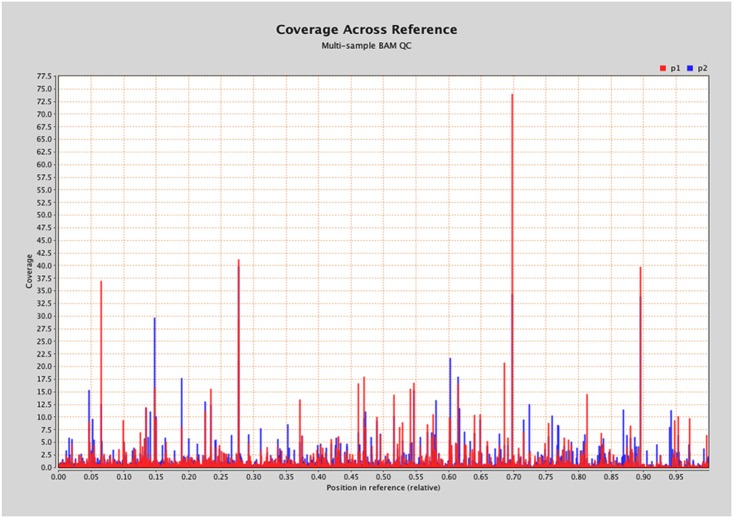
GBS tag coverage for P1 and P2 with overlap across P2 reference genome. P1 (red) and P2 (blue) GBS tag coverage overlap across the P2 reference sequence.

A primary goal in advancing genomic resource development and genome enablement for marker assisted breeding and trait association is to establish a system for high throughput genotyping. Here, we assessed the feasibility of using a restriction derived approach, such as Genotyping by Sequencing (GBS). Following the methods of Elshire et al., [16], the *Pst*I enzyme was used for genomic selection, followed by single end sequencing (with a multiplex design) on a HiSeq2500 (Illumina), which yielded 2.2M and 2.4M reads for Genotypes 1 and 2, respectively. Alignment of the raw reads of P1 to the P2 reference sequence resulted in 22,145 (P1), and 14,305 (P2) that aligned zero times, 1.6 M (P1) and 1.4M (P2) aligned exactly 1 time, and 855k (P1) and 799k (P2) aligned greater than 1 time (S4 Table). Next, these reads were collapsed into a representative set of unique ‘tagpairs’ for each genotype, where P1 had 69,898 unique tag pairs, and P2 had 62,974. These unique ‘tag pairs’ are representative of ~4.4M bp (0.84% of the P2 assembly) and 4M bp (0.76% of the P2 assembly) of P1 and P2, respectively. Alignment of the P1 tags to the P2 reference assembly resulted in a total of 16,340 tags (23%) that aligned 0 times, 45,008 (64.39%) that aligned exactly 1 time, and 8,549 (12.23%) that had multiple alignments for P1. The P2 reads aligned slightly better (as to be expected since it is essentially a self-alignment), where 9,066 tags (14.4%) aligned 0 times, 45,113 (71.64%) aligned exactly 1 time, and 8,794 (13.96%) aligned more than once (S4 Table). Overlap between the P1 and P2 GBS tags was relatively high such that 36,790 sites were overlapping allowing for a significant number of genotyping possibilities (Fig 3).

## Discussion

Enabling a crop for marker assisted selection is a critical goal or achievement in any contemporary plant breeding program. In most crop species, this is achieved through development of a set of markers (typically SSRs) and subsequently moving toward higher density marker systems (eg., SSRs or SNPs) on a genome-wide scale for understanding the genome complexity. Genome-wide marker systems can be applied in linkage mapping, association mapping, and establishing trait associations or QTL identification. However, it is often difficult to establish the translational interface between breeders, molecular tools and infrastructure, and apply these technologies in the field. Simple sequence markers have revolutionized the way plants are selected for, and are still a prominent technology in plant and animal breeding programs [23, 24]. Coding sequences are often a starting point for SSR discovery because of their high conservation and potential utility to be used across species [25, 26]. In this study, microsatellites (both EST-SSRs and genomic SSRs) with different repeat motifs (di-, tri-, tetra-, penta-, hexa-, and hepta-nucleotides) were chiefly observed. The results showed that majority of the repeat motifs were of di- and tri-nucleotide types, of which tri-nucleotide repeats were the most abundant one’s. This is expected considering the abundance of di- and tri-nucleotide sequences in other crop species [27] and tri-nucleotide repeats generally do not cause a shift in amino acid reading frame. In the EST-derived marker discovery pipeline, we observed a significant contrast in polymorphism rate when compared to the Genomic SSR pipeline, with a superior polymorphic rate of 83.51% in the Genomic SSR pipeline vs. 34% in the EST-SSR pipeline. This phenomenon of high polymorphism rate for genomic SSRs in comparison to EST-SSRs (identified from specific tissues showing their conserved nature) is also observed in other crops [28]. However, the genomic SSR method was designed to bioinformatically search for polymorphic SSRs which was not possible to perform with the EST data set. EST-SSRs also had a higher failure rate. It is also typical to observe a higher amplification failure rate when designing primers from spliced coding sequences, such as ESTs, where introns are difficult to determine and account for during amplification setup. However, the EST-SSR approach couples markers with genes, where the Genomic SSR approach yields markers randomly distributed throughout the genome, which in the long-term is a desirable outcome. From a discovery perspective, the total number of SSRs discovered in the Genomic SSR pipeline is far superior in the identification of candidate markers in addition to ease of experimental setup and technician time. From a cost and data collection perspective, it is hard to determine which approach is more desirable. Sequencing of the transcriptome through the EST-SSR pipeline is exponentially more cost effective than whole genome sequencing when just looking for genes, and with careful experimental design it could be coupled with an expression experiment to facilitate multiple data uses to increase impact for each dollar spent. As whole genome sequencing covers a much broader spectrum of the genome, more SSRs are anticipated to be discovered and the occurrence of polymorphism due to mutations does not face the overall same negative selection pressure as many mutations occurring within coding regions. Complementary to whole genome sequencing and genome-wide microsatellite marker discovery, is the ability to identify and analyze genetic variation at the discreet single nucleotide level. In that respect, Single Nucleotide Polymorphisms (SNPs) have readily advanced as the marker of choice in many model systems with high quality reference genome assemblies such as maize, sorghum, and rice. In non-model systems, such as yam, it is feasible to leverage the low cost of genome sequencing to determine SNPs within and between populations and utilize these markers on a genome-wide scale for association genetic analysis, trait elucidation, and ultimately marker assisted selection. Similarly, restriction derived genomic selection has facilitated unprecedented opportunities in genotyping large numbers of samples in a short period of time for a reasonable investment [9]. This technique could effectively be considered a disruptive techology with significant gains in modern agriculture of the 21^st^ Century [29–32]. In *D*. *alata*, we identified SNPs on a genome-wide scale by aligning the whole genome sequencing reads of the two genotypes, and determined the feasibility of utilizing a genotyping by sequencing approach for high throughput genotyping. Alignment of the genome-wide assemblies suggest a significant degree of polymorphism, as also determined by the microsatellite analysis.

With the ultimate goal of a cost effective SNP genotyping platform, we assessed the feasibility of using a genotyping by sequencing approach in yam by preparing GBS libraries of both genotypes and determining overlap of aligned tags to the P2 reference assembly. When establishing a restriction derived genotyping platform, choice of the genomic selection enzyme and sequencing depth is critical. The two most common enzymes in use are *Ape*k1, and *Pst*1. There can be a distinct trade-off between the number of sites sampled across the genome, versus depth of coverage. In species where a high quality reference grade genome assembly does not exist, low coverage sequencing, and marker imputation is not recommended. Rather, fewer SNPs and deeper coverage is recommended. Is this case, we used the *Pst*I enzyme for GBS, which in yam, is a less frequent cutter; presumably resulting in fewer sites represented, but allowing for greater sequencing coverage per sample. Our GBS efforts resulted in a significant number of genomic loci sampled, but more importantly, a high degree of similar sites that are in concordance between both genotypes sampled. Moreover, the genotypes can be discriminated at the GBS-SNP level, and data suggest that a *Pst*1 GBS technique is a powerful tool for dissecting closely related germplasm [33].

## Conclusions

The identification and selection of polymorphic SSR markers from ESTs or *de novo* assembled genomic DNA sequences, are both effective methods and can have immediate utility in molecular breeding. Overall, whole genome sequencing assures a better distribution of SSR motifs, avoids any unknown biases of standard SSR enrichment procedures and can provide genomic data that can be used for other purposes like SNP detection or gene discovery, but requires sequence depth at the expense of additional costs. EST-derived SSRs results in fewer markers with low polymorphism rate being conserved but can be a layer of a wider experimental design that can have overall more positive budgetary implications, at the expense of time in validating for polymorphism. In future studies, the inclusion of additional selection criteria, limiting a given contig (in the genomic SSR approach) to having only one SSR, it would assure a better distribution of SSRs across the genome; while multiple SSRs from longer contigs could serve as controls and corroborate linkage studies. In an effort to merge complementary marker types, we determined that the power of GBS-SNP genotyping is a suitable technology for high-throughput genotyping in yam. Our work has established a foundation for expanding and integrating these resources into a strategy for linkage mapping and genetic association to resistance to anthracnose disease, and many other traits in *D*. *alata*. Moreover, standards and best practices for combining different marker types and legacy data will have an impact on non-model species and step-wise genomic enablement.

## Supporting Information

S1 Table List of EST-SSRs, primer sequences, repeat motifs, product size and their polymorphism. (XLSX)

S2 Table De novo genome assembly statistics. (XLSX)

S3 Table Nucleotide base counts of the two genotypes and percent content. (XLSX)

S4 Table Details of genomic SSR data, repeat motifs, primer sequences, allele product size, and their polymorphism. (XLSX)

S5 Table Alignment of GBS representative tags from P1 and P2 to the P2 genomic assembly. (XLSX)
